# Prediction of Degraded Infrastructure Conditions for Railway Operation

**DOI:** 10.3390/s24082456

**Published:** 2024-04-11

**Authors:** Juan de Dios Sanz Bobi, Pablo Garrido Martínez-Llop, Pablo Rubio Marcos, Álvaro Solano Jiménez, Javier Gómez Fernández

**Affiliations:** 1 Department of Mechanical Engineering, Universidad Politécnica de Madrid-UPM, 28006 Madrid, Spain; juandedios.sanz@upm.es (J.d.D.S.B.); javier.gomezf@upm.es (J.G.F.); 2 Department of Applied Mathematics in Industrial Engineering, Universidad Politécnica de Madrid-UPM, 28006 Madrid, Spain; 3 Universidad Politécnica de Madrid-UPM, 28006 Madrid, Spain; rubio.marcos.pablo@gmail.com (P.R.M.); alvarosolano333@gmail.com (Á.S.J.)

**Keywords:** machine learning, flood risk management, predictive maintenance, railway dynamics, railway safety

## Abstract

In the railway sector, rolling stock and infrastructure must be maintained in perfect condition to ensure reliable and safe operation for passengers. Climate change is affecting the urban and regional infrastructure through sea level rise, water accumulations, river flooding, and other increased-frequency extreme natural situations (heavy rains or snows) which pose a challenge to maintenance. In this paper, the use of artificial intelligence based on predictive maintenance implementation is proposed for the early detection of degraded conditions of a bridge due to extreme climatic conditions. For this prediction, continuous monitoring is proposed, with the aim of establishing alarm thresholds to detect dangerous situations, so restrictions could be determined to mitigate the risk. However, one of the main challenges for railway infrastructure managers nowadays is the high cost of monitoring large infrastructures. In this work, a methodology for monitoring railway infrastructures to define the optimal number of transductors that are economically viable and the thresholds according to which infrastructure managers can make decisions concerning traffic safety is proposed. The methodology consists of three phases that use the application of machine learning (Random Forest) and artificial cognitive systems (LSTM recurrent neural networks).

## 1. Introduction

In recent years, sustainability and climate change have been two principal concepts to consider in society. Agenda 2030 contains a specific action plan for mitigating the consequences of climate change and correcting actions towards a more sustainable and equilibrated future [1]. Both fields are closely linked to urban and regional transport and are on governments’ daily agendas in all European countries.

Sustainability is being obtained through CO_2_ and other pollutants’ reduction, increasing the energy efficiency concept in urban transport, and focusing on a derivation of cargo and people transport from the roads to the railways, reducing traffic accident rates, increasing safety, and considerably reducing emissions to the atmosphere [2].

The EU’s 2030 strategy, aligned with the United Nations’ sustainable development goals, integrates transport in general and rail in particular as clear actors at stake. For this, actions are necessary in different sectors. Within the framework of the Horizon Europe research program of the European Union, Europe’s Rail Joint Undertaking will focus on digital innovation and automation to achieve the radical transformation of the rail system needed to deliver on the European Green Deal objectives. Standardization activities in CEN/CENELEC tackle the climate change challenges for railway application, making a deeper review of the environmental conditions due to climate change on the equipment used with rolling stock and on-board equipment (EN50125-1) [3], fixed electrical installations (EN 50125-2) [3], and equipment for signaling communication and coverage (EN 50125-3) [3]. This coverage of innovation activities and also the review of current standards raises a deep awareness of the impact of climate change on the preservation of the safety conditions of the rail system.

In the last years, an increase in extreme conditions has also been detected, and the consequences of these environmental behaviors could range from delays and lost trips to serious accidents due to water flooding or land subsidence [4]. In this paper, the use of artificial intelligence is proposed for the early detection of degraded conditions of a bridge as an essential element of railway infrastructure. Many tunnels, bridges, and other important constructions are monitored regularly by auscultation vehicles owned by infrastructure companies, but continuous monitoring of all those critical infrastructures does not exist. This constant monitoring has a very high impact on costs due to the sensors, electronics, and data sending required. In this paper, an optimization of the bridge sensing process is presented after a detailed study of signals measured during the passage of more than 20 trains over the bridge.

The novelty of this paper is the determination of a methodology that compiles the application of machine learning and artificial cognitive systems in the monitoring of railway infrastructures to optimize their maintenance, detect the presence of degraded conditions, and provide tools for infrastructure managers to make decisions during operation.

The methodology consists of three phases. The first one, based on the Random Forest algorithm, predicts the response at the abutments when a train is passing through them, and a significance analysis extracts the key points in terms of the health of the structure. The key variables to monitor are the abutment accelerations of the bridge (S1 sensor in the experiment), and it is demonstrated that relevant information is obtained from the central sensor located in the middle of the bridge span. The total length of the bridge does not affect the model, but the span length is an important parameter to consider. The second phase consists of the implementation of an ensemble of LSTM recurrent neural networks, which predict the response at each critical point detected in phase 2 that characterizes a normal dynamic bridge behavior (with no rain, water accumulation, or other extreme ambient or climate conditions). Finally, in phase 3, the methodology is implemented, during which the algorithm must be trained and define the error thresholds that will establish circulation restrictions. The methodology is evaluated on-site under real-world conditions. After the model optimization, three cases of heavy rains or water accumulation are introduced in the RNN to check the performance. The errors obtained are seven–eight times greater than the ones obtained with the model trained with data corresponding to good weather conditions. As there is a big difference in the magnitude order of the errors obtained, it clearly demonstrates the effects of river flooding and water accumulation on the bridge. This situation leads us to determine speed limitations in the cases where this degraded dynamic behavior of the bridge is detected (due to MAEs in the predictions greater than defined thresholds in several samples). A methodology for degraded case detection and risk mitigation due to river flooding or water accumulation is proposed.

All operators and infrastructure managers must ensure the safety of the entire rail infrastructure, and new technologies such as artificial intelligence used with all the signals measured could help us detect the principal hazards provoked by non-controlled extreme natural conditions.

## 2. Railway Approach

In the railway sector, safety has always been prioritized against other variables such as reliability or comfortability. It can be considered that digitalization is still at an early stage in rail transport. According to data from the European Commission for 2017, transport had an index of less than 15% in terms of digital intensity. When digitalization developed in the industrial and economic sectors, rail transport adopted it unevenly, with the rolling stock subsystem currently showing the greatest progress in digitalization. Nowadays, maximum levels of safety are technologically guaranteed with the on-board systems of the train and other safety architectures implemented in the infrastructure. For example, signaling systems continue to increase their safety levels by avoiding human errors and reducing potential risks [5]. But there are situations, such as natural disasters or extreme environmental and climate conditions, that have a clear impact on the infrastructure and that are considered a high risk to the train operation. Indeed, digitalization on the track, beyond signaling systems, has been limited. This is firstly because civil works have always enjoyed less digitalization than vehicles, both railway and road vehicles. This lack of monitoring represents a barrier to digitalization, driven largely by the economic cost of installing transducer systems with wiring, a problem that does not exist in a vehicle with a controlled environment. Currently, with the development of data management systems in the cloud and the implementation of faster communication networks, part of these problems have been solved, opening the door to new advances. Sensors placed on critical infrastructure points can send data that, once collected and processed, can detect defects or imminent breakdowns, presenting infrastructure managers with more possibilities. The current problem is not about establishing the fastest algorithms but rather the simplest ones that allow continuous monitoring. This simplicity is of great value due to the increasing importance of human factors in railway safety.

Concrete studies are being developed in order to improve infrastructure safety [6] in critical constructions such as tunnels, bridges, embankments, or railroad overpasses. In these examples, it is very important to control the climate conditions and to determine certain operational restrictions to mitigate any possible issues, such as speed limitations on high-speed trains on open-air bridges due to lateral crosswinds.

As far as monitoring is concerned, the main goal is to detect degraded infrastructural conditions, such as river flooding, water accumulation in bridges, or other infrastructural elements, in order to determine operational restrictions or service cancellations in extreme cases. To achieve this objective, it is necessary to obtain in advance information on the critical infrastructure status with trains passing. There are two different ways to face this problem: constant monitoring of the critical constructions (cameras, sensors) or minimizing sensing and creating a mathematical model based on a methodology capable of predicting and detecting degraded conditions of the infrastructure so that a mitigation plan could be implemented in real time. These prediction technologies are based on artificial intelligence algorithms such as Random Forest or neural networks [7,8].

In this paper, one specific bridge of the railway line Madrid–Cádiz is monitored in order to characterize and correlate the different measured accelerations of the bridge (which are directly related to vibration modes) when different trains are passing over it. Another important objective is to obtain a mathematical model capable of predicting the normal behavior of the bridge, so it could be possible to detect degraded behavior of the accelerations of the bridge by comparing the real data of a train passing with the predicted data. If there is a big difference between datasets, then degraded behavior is predicted. This difference will mean that additional efforts or forces are involved, such as water flooding, water accumulation at the base of the bridge, or unstable soil settlement. This degraded condition detection will allow the implementation of additional operational restrictions, avoiding train service cancellations and reducing train operation risks under extreme climate conditions. It is important to highlight that many techniques from the artificial intelligence family, specifically those related to the discipline of computational perception, are already being used in structural engineering. Indeed, frequency analysis for the identification of vibration modes, calculations of power spectral densities (PSDs), and windowing techniques are today essential for any dynamic structural study. All these techniques, preceding the analysis, are essential for the proper study of the problem, avoiding problems of aliasing or leakage. There are techniques based on stochastic subspaces (SSI) that identify the main dynamic parameters of a structure: modal deformation, modal frequency, and damping [9]. These techniques, known as modal analysis techniques, are used in the diagnosis of structures by evaluating the changes experienced by the continuous medium over time. Changes in structure are detected through variations in modal characteristics [10]. Notwithstanding, SHM is crucial for ensuring the safety and reliability of civil infrastructure. Traditional methods often struggle with the complexity of structural systems and the variety of data sources involved. With the emergence of machine learning techniques, significant progress has been made [11].

Consequently, regarding deep learning, its relevance in structural health monitoring is beyond doubt. These algorithms can obtain characteristics of a facility or structure from its behavior in dynamic situations by analyzing vibration parameters [12]. In this scenario, neural networks have attracted attention. However, one of the main handicaps in these studies is the uncertainties that surround the vibration measurements, which may lead to unreliable output results from such networks. Due to this, several studies have been undertaken to consider the effect of uncertainties in developing a neural network model [13].

Studies on these techniques intend to find a more adequate method to assess the structures. There are studies that address the classification of these techniques by comparing three widely used neural networks (artificial neural network (ANN), convolutional neural network (CNN), and long short-term memory (LSTM) network) [14]. LSTM neural networks have the capability to predict the response of a building at each point independently. So, they are adequate considering that time and frequency are widely used to extract features of the vibration responses because, as is known, the main concept of a recurrent neural network is the consideration of time steps; therefore, recurrent neural networks can be trained, regardless of the sequence length, on both the input and output layers [15]. Another important parameter in SHM is the remaining useful life (RUL), whose characteristics and long-term dependencies make the LSTM recurrent neural network useful in monitoring it due to its capability of handling complex temporal patterns [16]. This methodology has recently been used to predict comfort degradation [17] or bearing temperature anomaly detection in the context of condition-based maintenance as part of Industry 4.0 and maintenance [7,17].

Beyond deep learning models, machine learning algorithms can be applied successfully to damage identification problems. The principal challenge is to obtain enough data to train the algorithms properly, avoiding overfitting at the same time as building a robust model [18]. As far as the Random Forest model is concerned, this machine learning algorithm can be implemented so as to evaluate the health of the structure by analyzing the stiffness variations, where impulse responses functioning as structural vibration properties are extracted from the acceleration responses [19]. Other studies have shown that RF models can efficiently lead to a good damage identification result with much lower computational demand and time compared to neural network training methods [15].

From the point of view of optimizing the number of sensors, currently, part of the efforts is being directed towards the implementation of multi-agent systems [9], in which, based on the definition of roles and working groups, it is possible to obtain a better number of data combinations, which leads to distributions with fewer sensors that provide a reliable characterization of the state of the structure. There are also studies that integrate the SHapley Additive exPlanations (SHAP) method into an ML-based damage detection process as a methodology for assessing the contribution of each parameter in a prediction [20]. By understanding the relevance of each sensor in a model’s decision-making process, sensors crucial for accurate predictions can be selected, potentially reducing the number of sensors without sacrificing performance.

The state-of-the-art here discussed includes the different algorithms and techniques that make up the method this paper addresses. Nevertheless, the state-of-the-art regarding applied methodologies lacks proposals that integrate the methods together to obtain the advantages of each one and their synergies, leading to a methodology that optimizes the SHM in railway operations and is compatible with it.

## 3. Theoretical and Mathematical Foundations

In this manuscript, two main artificial intelligence algorithms are used: recurrent neural networks (RNN-LSTM), for the prediction of the measured accelerations in the safety-related points of the bridge, and Random Forest, for the characterization of the relationship between all measured variables in the bridge to determine the safety-related points (key points) used in the RNN algorithm.

The aim of the Random Forest algorithm in this method is to predict the accelerations of the abutment of the bridge, which is one of the most representative variables to characterize its normal dynamic behavior regarding safety. According to this, the column (feature) corresponding to the sensor S1 (placed on the abutment) will be predicted separately and afterwards compared with the real values to check the performance of the model. Therefore, this corresponds to a regression task in which one of the bridge’s accelerations is predicted in relation to the other variables in the dataset.

**Figure 1:**
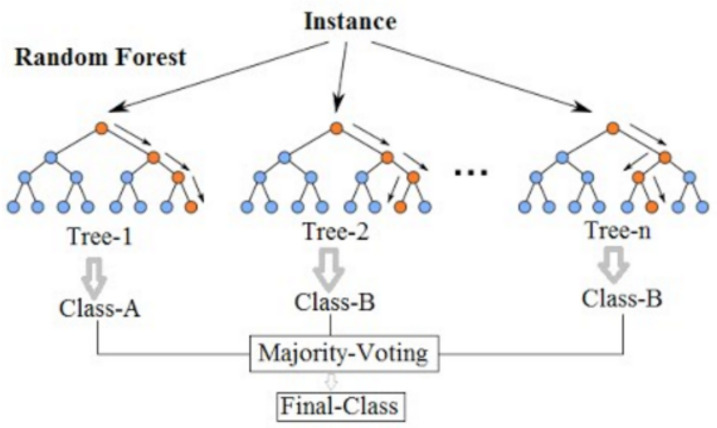
Simplified diagram of a Random Forest model.

A Random Forest machine learning model is chosen in the first phase of the methodology instead of a linear regression model since the acceleration ratio of a structure is not a linear problem. Indeed, the structural behavior depends on the points of application of the load and its frequency, and therefore better results are expected through the application of decision trees. Random Forest (RF) is one of the most accurate and widely used supervised learning algorithms for classification and regression tasks. Random Forest uses a random distribution of a predetermined number of decision trees, considering the average of all the results as the final value to be considered as a prediction. It is a case of ensemble learning. There is a hyperparameter in Random Forest models that determines the number of estimators (decision trees) that the model builds before taking the average of the results of these trees. In Figure 1, a simplified example of a Random Forest model and its different trees is shown.

A decision tree with p input variables works as follows:

- The p-dimensional feature space is divided into M mutually distinct regions that entirely cover a subset of the feature space and do not overlap.
- The value range of each variable is divided into two parts (nodes). The partition that minimizes the total error is selected, and the process continues, creating new nodes. This iterative algorithm ends once an acceptable error is determined. The function that is used to measure the quality of a split is usually the Gini impurity function, but sometimes the information gain is also considered.
- For the prediction process, a new case is pushed down the tree, and the label or number of the terminal node is assigned [10].

Random Forest presents fewer generalization errors and higher accuracy, which makes it appropriate for solving problems without prior knowledge or known relationships between input variables [21], and it has gained popularity due to its ability to handle high-dimensional data, non-linear relationships, and interactions among variables. In the context of SHM, these models excel at detecting subtle changes in structural behavior indicative of damage. In addition, traditional SHM systems often rely on fixed sensor configurations and sampling frequencies, leading to suboptimal resource utilization, whereas random forest models can analyze sensor data to identify critical monitoring locations and optimal sampling intervals.

Once the Random Forest model is adjusted, a SHapley Additive exPlanations (SHAP) algorithm based on game theory will evaluate the influence of each transducer in the final prediction of the model, with the aim of detecting redundant accelerometers and obtaining a minimum number of sensors that characterize the dynamic response of the structure. As a result, the safety-related points (key points) are identified, and at each one of them, a recurrent neural network is implemented separately. Deploying accelerometers for monitoring purposes involves significant costs and logistical challenges. By analyzing the impact of individual sensors on the model’s output, SHAP enables the identification of redundant or less-informative accelerometers. By leveraging SHAP to optimize accelerometer deployment, railway infrastructure managers can create cost-effective and efficient monitoring systems for bridges. These approaches ensure that resources are allocated judiciously, maximizing the quality and reliability of structural assessments [20].

After identifying the most informative accelerometers through the SHAP algorithm, a recurrent neural network is deployed at each accelerometer independently. A recurrent neural network is a deep learning algorithm capable of inferring certain behaviors, patterns, or relationships (especially non-linear relationships) from a given dataset of measured data. The architecture of the RNN is similar to that of an artificial neural network, formed by neurons and layers. Each neuron receives the values of the outputs computed by the neurons of the previous layer as an input and also computes the activation that will be sent to the following neurons.

Each neuron is formed by the following trainable parameters: w (weight) and b (independent term), and the inputs are subjected firstly to a linear operation and secondly to a non-linear operation:

$$i_{t}=\sigma \left(w_{i}\cdot \left[h_{t-1},x_{t}\right]+b_{i}\right)$$

$$C_{t}=\tanh\left(w_{c}\cdot \left[h_{t-1},x_{t}\right]+b_{c}\right)$$

$$f_{t}=\sigma \left(w_{f}\cdot \left[h_{t-1},x_{t}\right]+b_{f}\right)$$

$$o_{t}=\sigma \left(w_{o}\cdot \left[h_{t-1},x_{t}\right]+b_{o}\right)$$

$$h_{t}=o_{t}*\tanh\left(c_{t}\right)$$

where h is the hidden state and x is the input state.

**Figure 2:**
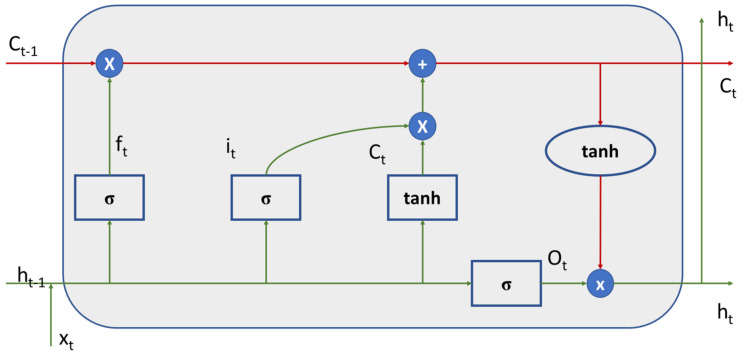
Simplified diagram of an RNN-LSTM.

The idea behind creating an RNN model is that it can learn and extract intrinsic relationships from the provided dataset, being able to generalize the normal behavior of the bridge’s accelerations or movements and use that knowledge to predict accelerations out of the normal range. Artificial neural networks predict results from their input layer. In this layer, all the characteristics of the model are introduced, and a prediction is obtained as the output. However, these architectures do not take this into account when predicting the output of the previous prediction. With RNNs, the prediction at time T considers the prediction at time T. This aspect is fundamental in dynamic systems, where the displacements, accelerations, and speeds are related to each other with differential calculation, and therefore each moment of time is restricted by the previous one. In addition, it is possible to use the minimum number of transducers, as it is a single, self-sufficient sensor. Decoupling the prediction between accelerometers allows a better analysis of outliers on the occasion of technical problems. Conventional RNNs present two problems: the vanishing gradient and the exploding gradient. This is produced by the fact that the backpropagation algorithm over time generates a chain of multiplications of values between 0 and 1, which means that the network stops learning. On the other hand, the “forgetting” of the predictions generated with large temporal spaces occurs. This condition leads to the proposition of a long short-term memory (LSTM) architecture, which is represented in Figure 2. 

This architecture introduces a series of doors (“gates”) in the RNN that determine what information is retained and what information is forgotten from the information generated in each step [9]. These gates generate algebraic operations of addition and subtraction instead of multiplication, which mitigates the problem of the vanishing gradient and the exploding gradient.

Firstly, trainable parameters are randomly initialized with a standard normal random distribution of zero mean and unit variance. For better performance in this paper, the normal initializer is also applied [22], which is commonly used for ReLU activation functions. Neural networks are iterative models; for the first iteration, a random prediction of the desired variables is obtained. Random prediction of the desired variable (accelerations in this study) is obtained, and the error value is quantified through a cost function, which is the objective function of the computational model. The cost is computed by comparing the predictions to the real values (target values). New iterations are performed, evaluating the cost function gradient to reduce the error value and updating the parameters on the “backward propagation” algorithm. Finally, the optimized parameters are obtained, and the cost function is reduced to the minimum, as are the measured errors.

A structural system is modeled by a set of dynamic variables that reflect its inherent characteristics [23]. These characteristics—stiffness, mass, and damping—will determine the behavior of the bridge. A system modeled this way can be considered a black box and is invariant in normal operation (weather conditions that are not adverse and no sudden structural degradation). As a result of the comparison between the prediction and the measured values, metrics are obtained. Changes in the system are due to the following:

- Premature degradation of the structure with a change in stiffness.
- Change in boundary conditions due to earthquakes and heavy rains that generate changes in damping.
- Mass changes. They can occur on very small viaducts due to floods.

As mentioned, the system can be considered a black box. This is because if the inherent characteristics do not change, the same response is obtained under the same load. Consequently, each RNN will generalize the black box; that is, it will learn the inherent characteristics of the infrastructure. From the first recorded accelerations, the system predicts the expected response. If the metrics offer poor values, it is an indicator that the prediction and the response are not equivalent and, therefore, the behavior of the infrastructure is not under normal operating conditions. This is an indicator that the asset is in a degraded situation, and decision-making should be assessed accordingly.

## 4. Problem Description and Measured Data

**Figure 3:**
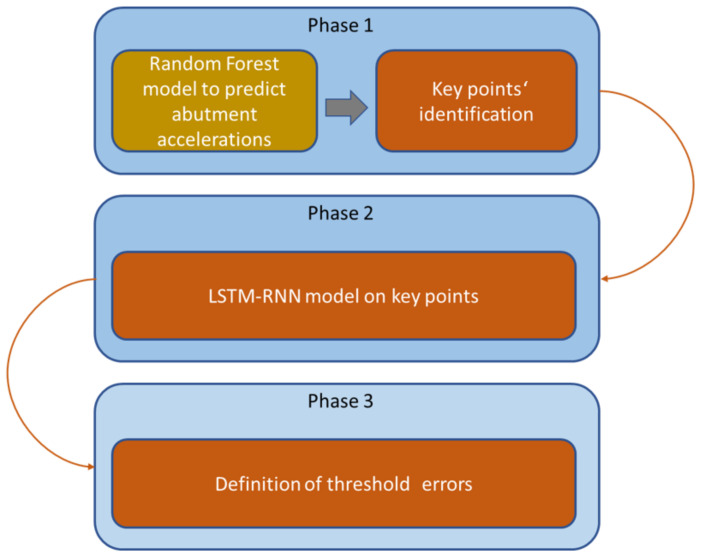
Methodology flow chart.

In this paper, a methodology to define the optimal number of transductors that are economically viable and the thresholds according to which infrastructure managers can make decisions concerning traffic safety is proposed. The methodology comprises three phases, as illustrated below in Figure 3, each one with a specific objective. The work proposed is prior to operation, as the phases developed in this work culminate with the commissioning of a viaduct monitoring system whose predictions will support decision-making related to the maintenance and operation of the infrastructure. However, in addition to the procedures related to the management of the company’s digital maturity and organizational and procedural changes, the implementation of technology must be managed.

**Figure 4:**
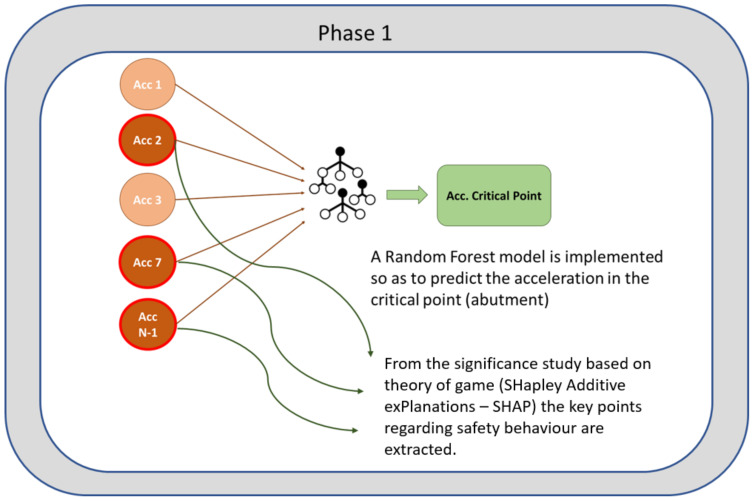
Phase 1 of the methodology.

The first phase of the methodology consists of two parts as it is shown in Figure 4. In the first part, a Random Forest algorithm that predicts the response at the critical points (abutments in the case of viaducts) is developed [11]. Based on this algorithm, in part two of phase 1, the points with the greatest influence on the prediction are analyzed using the SHAP algorithm [20]. These points will be those that define the state of the infrastructure.

**Figure 5:**
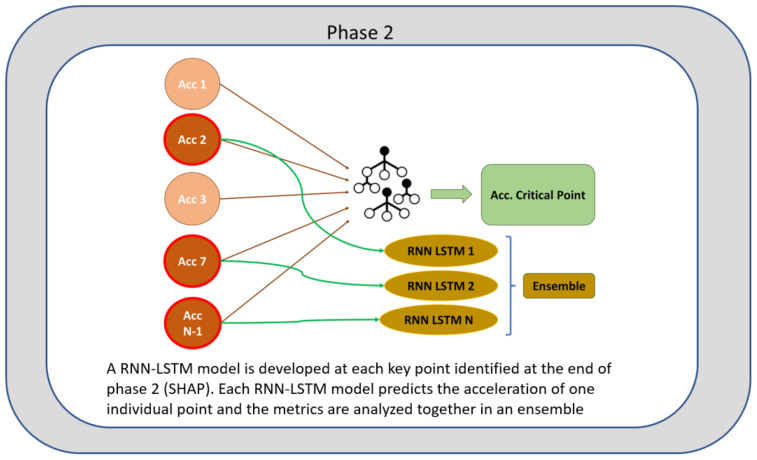
Phase 2 of the methodology.

In phase 2, represented in Figure 5, the points that characterize the structure from a safety point of view are identified. The accelerometers identified in the previous section can predict the response at a critical point in the structure. However, some depend on others, and reducing the number of accelerometers involved in the prediction decreases precision. In this section, an LSTM RNN is created for each independently identified accelerometer, as illustrated below.

In phase 3, the methodology is implemented, a period during which the algorithm must be trained and define the error thresholds that will establish circulation restrictions. Every intelligent algorithm must generate a model that generalizes the solution, thus avoiding overfitting. To do this, it must be trained with a large amount of data that reflects the real universe in which the technology will operate. After training, alarm thresholds must be established based on the selected metrics. These thresholds are critical when implementing the methodology since they will serve as triggers when launching warnings. In the previous section, algorithms based on deep learning were generated that predict the response of the structure at the points that have the greatest influence on the dynamic behavior of the critical point of the infrastructure identified in phase 1 (Random Forest). The advantage of this is that each LSTM RNN predicts the outcome at one of these critical positions independently, and the error is evaluated. When a significant error occurs that exceeds what is expected, it may be due to several factors that boil down to two issues: a measurement error or a change in the characteristics of the structure.

**Figure 6:**
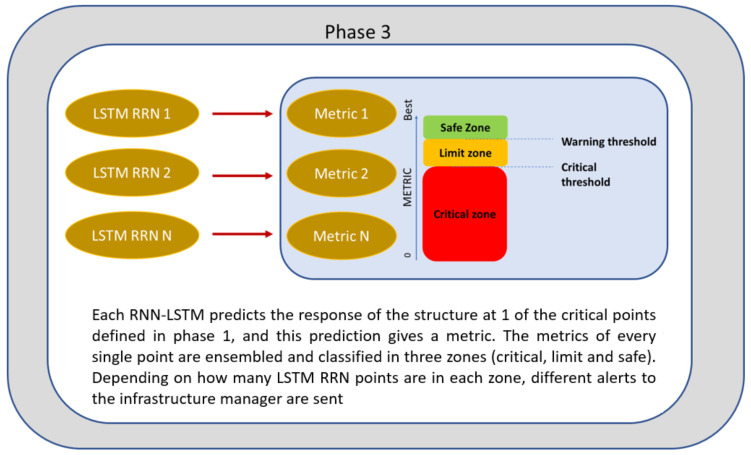
Phase 3 of the methodology.

After training the networks, a voting system is established that monitors the state of the structure based on the errors of each LSTM RNNs. This error places each prediction in a zone: the safe zone, the limit zone, or the critical zone. This is represented by Figure 6.

As the methodology proposed in this work consists of an ensemble of independent LSTM RNNs, the measurement error is easily discarded through a voting system. If all the predictions are correct except one, due to structural engineering, it is clear that there is an error in the registration since the response of a structure responds to a series of dynamic characteristics that prevent this randomness. For these reasons, at least the two most important points extracted from the SHAP algorithm must be installed in operation.

When the prediction error is widespread, we are faced with a change in the structure that poses a security risk. These changes can be linear or non-linear and are associated with alterations in the rigidity of the infrastructure or climatological effects.

In this manuscript, a specific bridge of the railway line Madrid–Cádiz is studied. Heavy rains incur two main serious consequences for this bridge: water accumulation on the surface of the bridge where the rails are fixed and a sudden increase in the river flow that crosses under the bridge, which could cause river flooding and, in both cases, very dangerous situations for normal train operation.

An experiment was carried out in 2005 to determine the vibration mode of the bridge and other technical characteristics such as stiffness or damping parameters.

**Figure 7:**
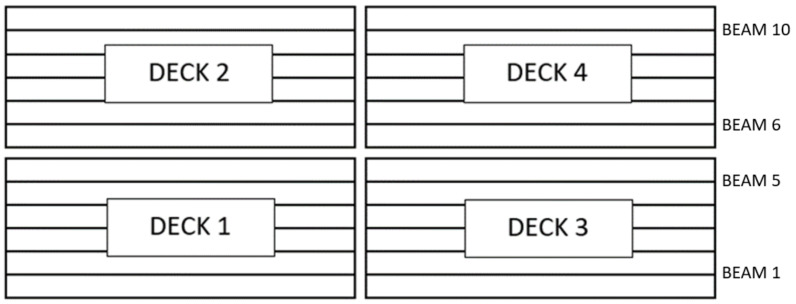
Principal structure of the bridge.

The bridge consists of a single central support and two lateral supports (abutments). The deck is divided into four independent sub-decks (one for each direction of traffic and span). Decks 1 and 3 correspond to one track, and the other two to the other track. This is represented in Figure 2. The study focused on one of the spans, with deck 1 being instrumented on the first day and deck 2 on the second day. The response of the bridge to the passage of trains in both directions of traffic was observed. In Figure 7, the different parts of the bridge are described. 

**Figure 8:**
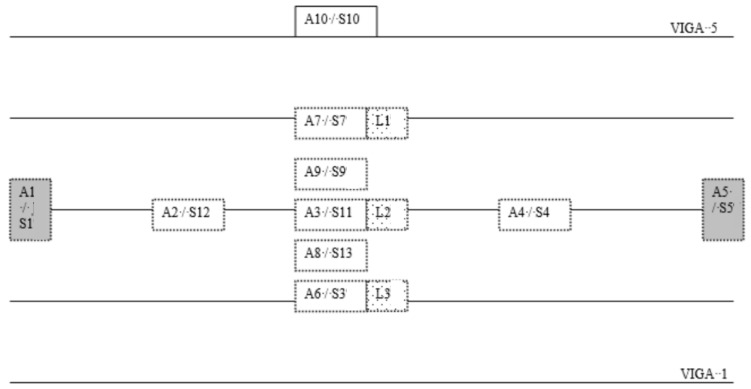
Diagram of the installed sensors.

For a good characterization of the accelerations of the bridge with different trains crossing over it, a set of transducers was installed on the bridge for recording. Two types of sensors were used: accelerometers and laser distance sensors. The accelerometers were placed on the central beams of each deck, on the deck, on the abutments, and on one of the sleepers of the track under study. The sensors were fixed with adhesive material and in a vertical orientation. The laser sensors were placed at the mid-span point of each of the central beams. A representation of the transducer’s location could be found in Figure 3, where the acceleration and laser sensors are located under the bridge except for number 10, which is located in one railway sleeper. Sensor 2 was placed next to S13 (redundant information), so only S2 data are considered for the prediction. S6 was placed on beam 1, but, as the reliability of the measurements was low, the data were removed from the prediction model. Sensors are represented in Figure 8. 

**Figure 9:**
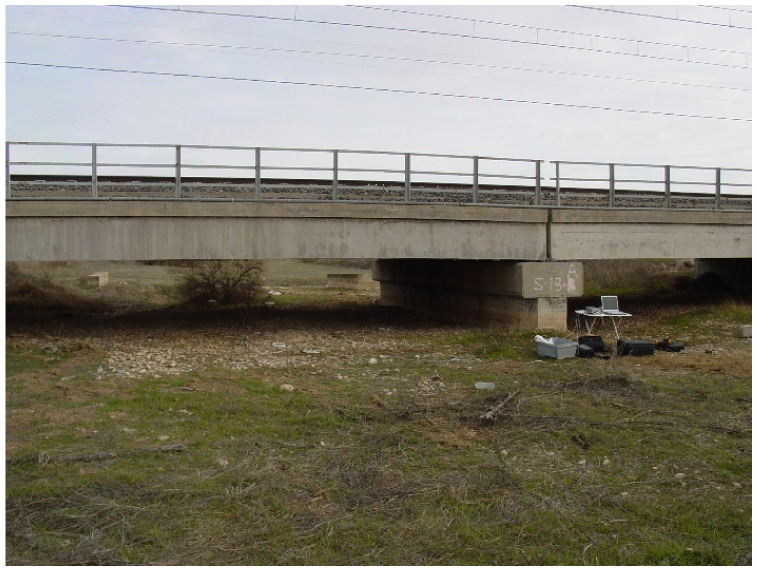
Instrumented bridge studied in this paper.

The position of all the sensors was previously agreed, according to the results of the dynamic study of the facility. As can be seen in Figure 9, the height of the bridge is very low, and for this reason, it is very important to determine if river flooding occurs.

The transducers deployed on-site are divided into two groups: lasers and accelerometers. The first ones aim to register the displacement of the bridge, and their purpose is to check the data registered by the accelerometers by a comparison between displacements and accelerations, thus guaranteeing the physics relationship. They have two working modes. The first is in high resolution, offering a measurement precision of 8 μm (resolution mode), and the second is in high speed, responding in times less than 660 μs (speed mode). In this project, the sensors were configured in resolution mode, seeking to characterize the deflection of the bridge with the highest possible resolution. The accelerometers installed are capacitive with a digital output (PWM) based on an integrated circuit, ADXL213. The device is powered with direct voltage (3–5.25 V) and reacts to any excitation by offering the acceleration of movement in a digital format (square signal with the information contained in its work cycle). Table 1 below compiles the main characteristics of the instrumentation.

Regarding signal acquisition, each sensor is connected to a transmission conditioning module responsible for regulating the supply voltage of the transducer and converting its response to be transmitted by optical fiber. The signals arrive at the reception module, where they are again converted into analog signals, which in turn are captured with a data acquisition card.

A total of twenty-five complete train passages over the bridge were registered, of which three of these situations corresponded with rainy days. The train typologies were different, consisting of cargo trains, commuter trains, and long-distance trains. This ensured that we obtained a general conclusion for all the train typologies and that the model would not be particular to specific train units.

The complete dataset that is used in this manuscript includes the 22 normal cases that do not correspond to heavy rains or other extreme climate conditions. As occurs in every prediction problem, a target variable is needed. In this case, after expert consultation, the main variables that could help to detect a real problem in the bridge due to river flooding are S1 and S5, which are the acceleration sensors placed in the lateral abutments of the bridge and it are physically linked to the bridge surface but also to the soil and sub-soil that support the complete bridge structure.

Using the described dataset, the methodology proposed is developed in this study as follows:

1. Relation between the measured variables: Applying the Random Forest algorithm, the relation between all measured variables is obtained. It is essential to know the exact relation between the different accelerations of the different parts of the bridge to determine which are the most important to characterize the movement of a bridge under normal conditions (not heavy rains or other extreme climate conditions) when a train is passing over it and consequently to optimize the number of transducers needed [20]. It is also important to understand if there are representative variables that should be monitored for safety reasons.
2. Prediction of degraded conditions: By applying an ensemble of RNN-LSTM in the key points identified in the Random Forest and game theory algorithms, a predictive optimized model is obtained to determine the normal behavior of the bridge accelerations when a train is passing through them. If the error determined by this model starts increasing with different consequent train passages, it will mean that additional efforts or forces are occurring, and in the studied bridge, this will mean that there is a strong water influence via the river flow, river flooding, or even water accumulation on the surface of the bridge.

To evaluate the performance of each model, two performance metrics are defined: the mean absolute error (MAE) and the root mean square error (RMSE). Both indicators are commonly used in the state-of-the-art.

## 5. Data Cleaning and First Analysis

In the first step of the methodology, before starting with the artificial intelligence algorithms in phase 1, there is a previous important step that needs to be completed: the data cleaning process. A good and robust dataset is essential to obtain faithful and reliable results. In this sense, a first study of the data is performed to obtain a final global dataset ready to be used for the algorithms. The initial conclusion from the dataset should be that all accelerations follow a normal distribution centered at 0 with a very small standard deviation.

All outliers and atypical data are removed, so a homogeneous dataset is obtained. Measured data are robust enough in general, so less than 10% of the data is eliminated. There are no equal inputs (duplicated rows in the dataset), which is expected as there were not two equal trains passing over the bridge at the same time and with the same ambient conditions (temperature, humidity, etc.).

The final size of the dataset without the rainy cases (22 trips) and after the cleaning process contains approximately 20.000 rows.

**Figure 10:**
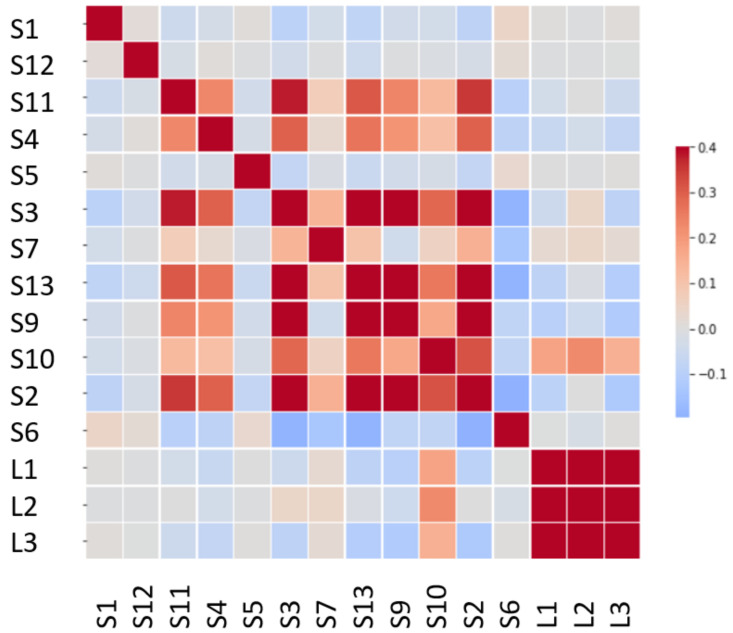
Heat map of the variables’ linear correlation.

A heat map for the data correlation is represented in Figure 10. One important conclusion is that the laser sensors and acceleration sensors are not highly correlated, so they apparently represent two different measurement procedures and do not give additional valuable information.

The study of the correlation matrix is based on the foundations of the dynamics of structures. The vibration modes of a structure are orthogonal to each other, which supposes an independence between their spatial shapes. This is reflected in the matrix, where the accelerometers whose main mode shape corresponds to different vibration modes show a low correlation to each other due to this fact. On the other hand, accelerometers located in antinodes of the same mode show a higher correlation (>0.5).

After global analysis and cleaning of the data, the artificial intelligence algorithms are applied, as detailed in the next chapters.

## 6. Random Forest Model and Results

**Figure 11:**
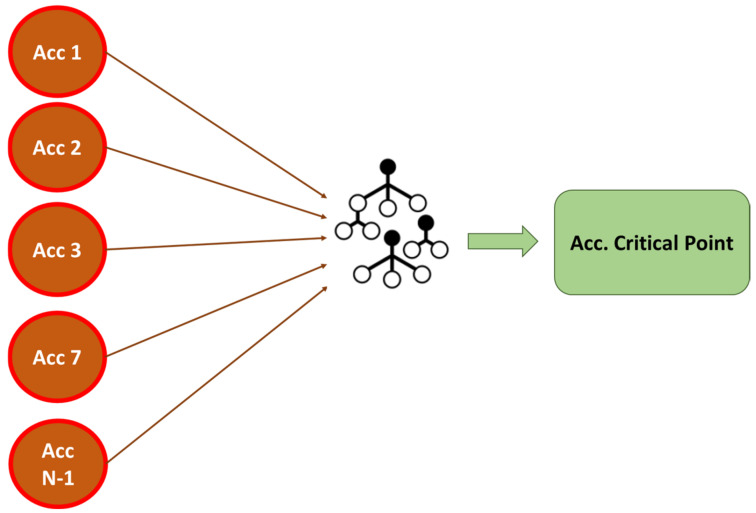
Phase 1 of the methodology (Random Forest model).

The first phase of the methodology consists of developing a Random Forest regression model to predict the structural response at a critical point of the structure (the abutment). Then, the SHapley Additive exPlanations (SHAP) algorithm is implemented to evaluate the influence of each transducer in the final prediction of the model, with the aim of detecting redundant accelerometers and obtaining a minimum number of sensors that characterize the dynamic response of the structure, and therefore optimizing the deployment of transducers. These accelerometers will be used later in phase 2 to develop a predictive model based on recurrent neural networks (RNNs). In Figure 11 the methodology of phase 1 is represented. 

The hyperparameters of the model are adjusted based on the evaluation of the out-of-bag (OOB) error. In bagging methods such as Random Forest, part of the observations of the dataset is not used to train the algorithm. These observations that are left out of the development are subsequently evaluated by obtaining the OOB error. In this work, the OOB error is evaluated using the R2 metric for different numbers of features and trees.

**Figure 12:**
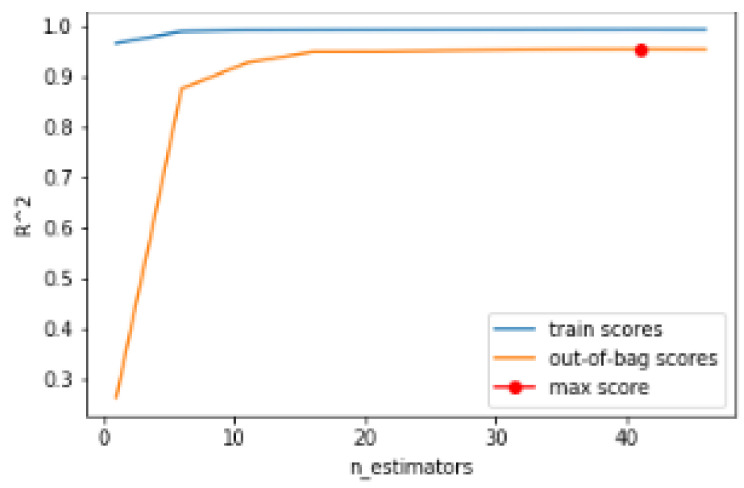
Number of trees assessment.

Regarding the number of trees, 42 is taken as a hyperparameter because it is the average obtained after the simulation of the available data set. The number of trees assessment is represented in Figure 12.

**Figure 13:**
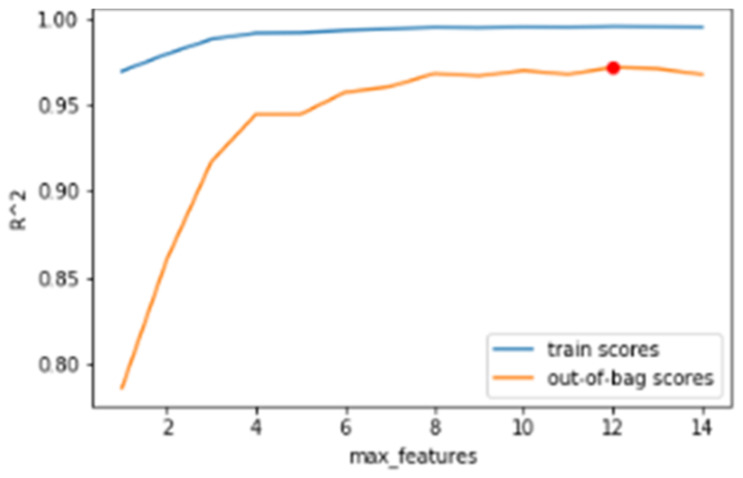
Number of features assessment.

Regarding the hyperparameter of the number of features, the analysis is carried out, obtaining an average of 12. Since there are a total of 15 transducers and there is not a big difference between 12 and the number of features available, the algorithm always takes the maximum number of available acquisition channels. This is justified, in addition to the little advantage in reducing the number of features by one or two, in that sometimes not all fourteen can be available due to a failure in one of them. This is represented in Figure 13. 

As stated before, an important goal of this paper is to obtain the relation between the different measured variables. In the data registration, many variables (and sensors in the end) are involved, but it is interesting to know the minimum variables needed to obtain a clear picture of the behavior of the bridge in terms of accelerations and, consequently, the vibration modes. The group of SHAP values demonstrates how much each predictor contributes, either positively or negatively, to the target variable fixed. As a conclusion, the higher the SHAP values are, the greater the influence the variable has on the target value. These accelerometers will be used in phase 2 of the methodology to generate a recurrent neural network bagging algorithm that detects the presence of anomalous behaviors of the structure. Based on this algorithm, the points with the greatest influence on the prediction of the critical points (abutments) are intended to be used to predict the structural response in a decoupled way by implementing a recurrent neural network (RNN) at each point. Finally, the conclusions of each RNN prediction of each point are ensembled by a voting algorithm to assess the structural health.

In Table 2, a diagram with the importance of each variable in predicting the target value is represented. The target value to be predicted is S1, as explained above, because it is placed in the lateral abutment of the bridge.

For the prediction of accelerations by S1 (left lateral abutment sensor according to the diagram in Figure 3), the variables that are most influential correspond to sensors S2, S7, and S8. This means that the variables that most explain S1 are located on one side of the central part of the monitored deck (in the middle, between the left abutment and the central pillar of the bridge).

As a general conclusion for this study, by only placing two sensors in the antinode of the first and second mode shapes of each span of the bridge, it is possible to determine the principal dynamic behavior of the bridge considering the effects of the river, terrain (sub-soil and soil), and other boundary conditions. All the other input variables have a low influence and explicability in the global dynamic behavior of the bridge. For this reason, the monitoring of this dynamic behavior could have been executed with these two sensors.

## 7. RNN Model and Results

**Figure 14:**
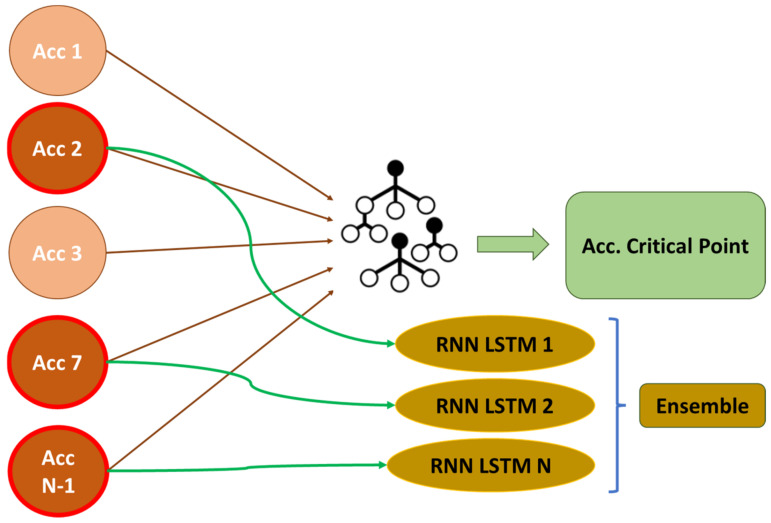
Concept of phase 2 of the methodology. Ensemble of RNN-LSTM.

The machine learning regression algorithm implemented in phase 1 predicts the response of the abutment using data from all the accelerometers installed. After this prediction, the SHAP algorithm inferred the positions with the greatest impact on the prediction of the critical accelerations and therefore had responses with the greatest influence on the safety of the structure. In this phase of the methodology, a recurrent neural network (RNN-LSTM) for each accelerometer is developed. The structural response is predicted for each of the previously identified points individually, and the error is calculated by a voting system. This is represented in the following Figure 14. 

An LSTM recurrent neural network allows the prediction of the time history of a signal from an input. Therefore, with this algorithm and a single accelerometer, the response of the viaduct at that point can be predicted. However, in operation, safety should never be left to the reliability of a single sensor. To avoid this, there are two alternatives:

- Make the accelerometer redundant. If a sensor fails, a second back-up transducer is available. This has several disadvantages. The first is that when the measurement failure is due to a defect in the region due to causes such as falling objects, it is likely that not only one accelerometer will be disabled but both. The second disadvantage is that two accelerometers are used, and information is only obtained from one point.
- Place accelerometers at critical points of influence detected in machine learning. With this system, the chosen one for this proposal, accelerometers are placed at the critical points identified in machine learning, and each one predicts the response at their position. That is, unlike Random Forest, each accelerometer only generates data for one recurrent neural network, and as a result, as many networks as accelerometers are implemented. In the event of an error in one of them, if the error does not occur in the other point, a degraded state of the infrastructure can be ruled out and measurement errors can be identified. The advantage of this model is the fact that it collects data from different points and avoids associating measurement errors with geometric failures.

According to the argumentations above, three RNN-LSTMs are built in order to predict the accelerations of S2, S7, and S8 identified in phase 1. The activation function used in the hidden layers is the ReLU activation function, and in the output layer is the identity function. The parameters are initialized with Glorot normal initialization, and the RNN also uses Adam optimization to train. The architecture of this RNN-LSTM is represented in Table 3. 

The three RNNs are trained, tested, and validated with the 22 train passages and the cleaned data with the purpose of predicting their own structural responses, which are the key accelerations to monitor and control, as explained above, due to their influence on the abutments’ behavior.

The dynamic response is characterized not only by the values obtained but also by the frequency content. Indeed, when designing a structure, the fundamental parameters are its natural frequencies. Therefore, the goodness of the prediction cannot be confined exclusively to point-to-point comparison but to its form in the frequency domain. The proposed network uses the window method. In this method, the time history is divided into windows of equal length, where the training batches are generated. These windows are shuffled to obtain a “bag” of training data. This split is performed randomly and only with data that are considered to be following “normal behaviour”, which means that they do not correspond to flood periods. In this way, the RNN will only learn to infer the normal behavior of the bridge. The train set contains around 20,000 instances, and the test set contains around 3000 instances. As for the size of the moving windows, given the importance of frequency identification, this must be greater than at least twice that necessary to obtain a frequency resolution of 0.5 Hz and a Nyquist frequency of 50 Hz in such a way that all the frequencies that are identified in the signal acquisition can be trained.

After adjusting all the hyperparameters and parameters of the model, the results are presented in Table 4.

The performance key indicator that should be used for this particular problem is clearly the MAE. Further, the error %, the important factor in this case, is to determine when the bridge is not behaving in a normal way, so precision in the predicted value is not as important as it is to know the general resolution of the model results. In this case, it is concluded that this model is able to predict accelerations with an MAE under 20%. So, a limit could be established in order to detect an early degradation of the dynamic behavior of the bridge. This limit will be suggested in the final conclusions.

On the other hand, it is important to consider that this model could be extrapolated to other bridges, as the study is based on one module (deck) between two main bridge pillars. In case a bridge is composed of “N” pillars, the same methodology could be applied and should work with similar precision levels. Other values of the accelerations will be obtained (as they depend on the material, length between pillars, and other boundary conditions), but the methodology is the same.

**Figure 15:**
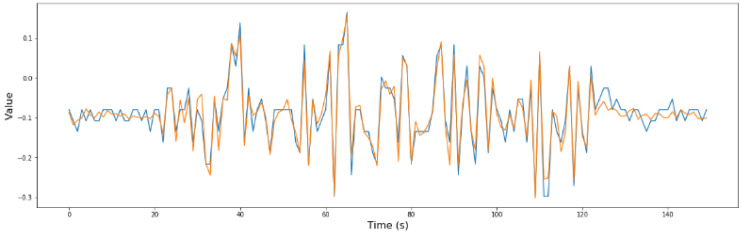
Real values (blue) vs. predicted values (orange) in the optimized RNN model of S2.

**Figure 16:**
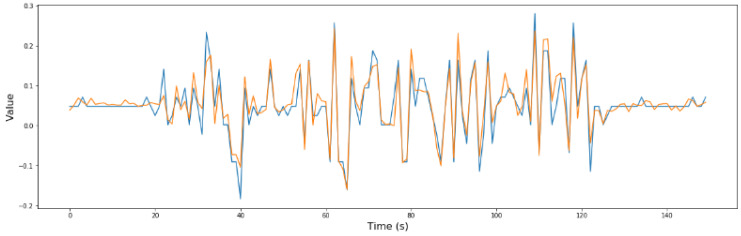
Real values (blue) vs. predicted values (orange) in the optimized RNN model of S7.

**Figure 17:**
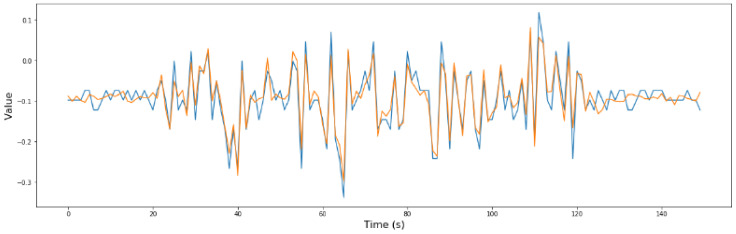
Real values (blue) vs. predicted values (orange) in the optimized RNN model of S8.

From the previous phase, phase 1, it was determined that sensors S2, S7, and S8 are critical for security. For a better understanding of the model, in Figure 15, Figure 16 and Figure 17, the comparison between the predicted values and the real values is shown.

As the methodology proposed in this work consists of an ensemble of independent RNN-LSTMs, the measurement error is easily ruled out by means of a voting system. If all the predictions are correct except one due to structural engineering, it is clear that there is an error in the registration since the response of a structure responds to a series of dynamic characteristics that prevent this randomness. When the error in the prediction is widespread, we are dealing with a change in the structure that poses a security risk. These changes can be linear or non-linear and are associated with changes in the rigidity of the infrastructure or with weather effects such as natural disasters related to river flooding or water accumulation. This section proposes an MAE threshold from which limitations on rolling stock circulation (speed or even cancellation of circulation) should be proposed by calculating between voted MAEs.

It is proposed to establish three areas composed of two thresholds:

- Threshold limit. When the metrics are above this threshold, the infrastructure is in a healthy state. In cases of metrics below this threshold but above the critical threshold, the measurement is in the limit zone. We propose this to be 0.7 of the best value of the metric obtained in the validation of the algorithm, or 1/0.7 of MAE.
- Critical threshold. When the predictions show values whose metrics are below this threshold, the structure will be in a critical zone. We propose this to be 0.85 of the best value of the metric obtained in the validation of the algorithm, or 1/0.85 of MAE.

**Figure 18:**
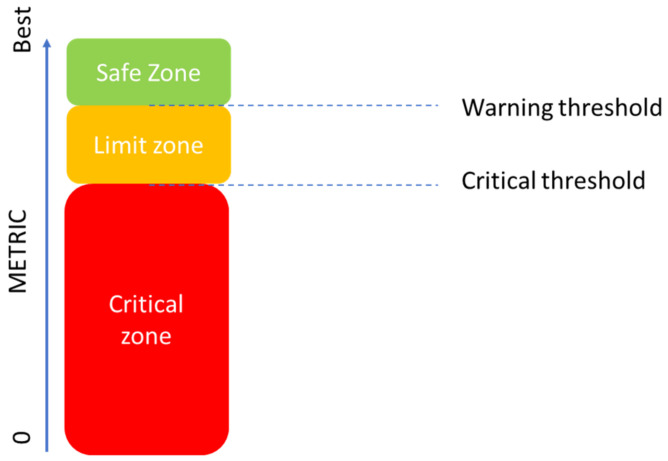
Metric threshold and areas proposed.

The proposed criteria depend on the number of predictions with MAE above the proposed thresholds, as it is shown in Figure 18. 

Safety criteria proposed is shown in Table 5.

The phases proposed here culminate with the operation where, based on the predictions obtained from the ensemble of LSTM-type RNNs, decisions are made regarding the maintenance and operation of the infrastructure.

A better result of the model is very difficult to achieve due to the nature of the data (acceleration sensors mainly) and that not so much data are available (only up to 20.000–30.0000 data points, considering that the performance of the model is highly related to the number of data points contained in the dataset). Therefore, for the construction of the models and the validation of the systematics, an implementation process is necessary, as occurs with other devices related to safety, such as axle counters. In this case, in addition to the causes associated with the reliability and field validation of the device, an implementation phase is necessary to generate a dataset that is robust in all cases that may arise.

This paper proposes a 12-month implementation where the models will be adjusted and based on which the thresholds described will be defined for each monitored infrastructure. During these 12 months, it is important that the registered data are compared with the data of the vehicles that circulate, in coordination with the circulation areas.

**Figure 19:**
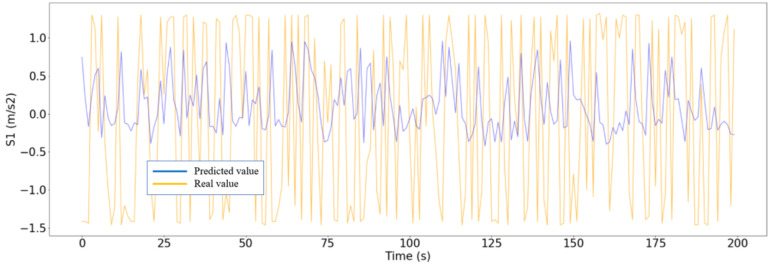
Difference between the predicted values of S1 (blue) and the real values (yellow).

The RNN developed data from the three trips in which the rain and the river flow influenced the bridge’s dynamic behavior, which were tested to verify if the model is able to obtain greater errors (degraded dynamic behavior detection) or not. After introducing these three cases, the errors obtained by the model were extremely high, so a degraded behavior of the bridge could be detected. In Figure 19, one of these situations is shown.

The comparison between the RMSEs and MAEs is found in Table 6. In this table, a limit value is also proposed to determine if the influence of the water accumulation or river flow started to affect the dynamic behavior of the bridge in order to limit the speed of the trains during the period of time that the infrastructure is affected.

In this section, the optimized RNN-LSTM is presented for the prediction of the degraded behavior of the bridge in terms of the accelerations measured. As the RNN is trained with data corresponding to normal dynamic behavior of the bridge, once data corresponding to not normal behavior are entered into the model, the algorithm presents very high errors. So, even the MAE or RSME factors are perfectly able to detect abnormal dynamic behavior of the bridge. In this sense, limit values are proposed to trigger an alarm, so the infrastructure manager responsible could make a decision to enable special measures for safety reasons such as speed limitations in the train operation or train operation cancellation.

## 8. Conclusions

In this work, a methodology for the prediction of degraded conditions in infrastructure (a bridge) is proposed. The results of the prediction are good enough to allow the infrastructure manager and operator to implement in advance different actions for mitigating the risk. With this, the most reliable and safe operation of the rail service is assured. Additionally, artificial intelligence techniques are used to optimize the number of sensors to be used for this purpose, reducing costs and increasing efficiency.

The methodology consists of three phases:

- Phase 1: A Random Forest algorithm predicts the response at the abutments (critical points) when a train is passing through. A significance study extracts the key points to be monitored with the aim of characterizing the structure’s health.
- Phase 2: The response at each critical point detected in phase 2 is predicted in a decoupled manner and trained with no rain, water accumulation, or other extreme climate conditions. Contrary to the Random Forest model, each accelerometer only generates data for one recurrent neural network, so an individual RNN predicts the values of each singular accelerometer.
- Phase 3: The methodology is implemented on-site. In this phase, the algorithm must be trained and define the error thresholds that will establish operation restrictions.

The evaluation of the methodology is carried out through on-site test campaigns where different typologies of trains pass over a bridge. In some cases, the operation is conducted under degraded conditions that could increase the risk of incidences. From these data, good results are obtained in the application of the first phases of the methodology:

- It is possible to predict the structural response at a critical point of the structure using the Random Forest algorithm.
- Using the Random Forest algorithm, it is possible to obtain the key points of the structure regarding the evaluation of structural health (SHapley Additive exPlanations).
- It is possible to predict the response at key points in a decoupled manner thanks to LSTM-type recurrent neural networks.

After the data analysis and from a mechanical point of view, there are no highly correlated linear variables. It is also determined that the most relevant accelerations to monitor and control are the abutment’s accelerations, so this is the main target for the prediction. There is no direct relationship between sensor 10 (in the rail) and the rest of the sensors, which can be expected as it is the only sensor above the bridge and not under the bridge.

From the Random Forest algorithm, the relation between the different measured variables (accelerations mainly) is obtained. To predict the abutment accelerations (S1), the most relevant sensors that can better explain these target variables are those located in the center of the span of the bridge, between the abutment and the central pillar. As it is clearly shown from S1’s prediction, only one sensor in this central position could be enough for a good explanation of the target variable and then for the dynamic global behavior of the bridge without heavy rains, water accumulation, or other extreme boundary conditions. The most simplified schematic should contain the abutment’s sensors and a central span transducer. As the bridge is formed by “n” modules and length is not a relevant variable in the study, it is concluded that the total length of the bridge does not affect the outcome but that the important variable is the length of the spans.

An RNN-LSTM is created and optimized for the prediction of the normal behavior of the bridge studied. The errors obtained by RMSE and MAE are 88.02% and 37.46%, which are not very low, but for this application, the most important issue was to obtain a controlled error in order to compare it with other situations where ambient conditions (rain, water accumulation) have an influence. The RMSE may not be the best key performance indicator due to the higher errors, so the MAE is selected as the appropriate key performance indicator (KPI).

The results obtained in extreme climatic conditions (heavy rains) lead to errors of several orders of magnitude higher than the model evaluated under normal operational conditions. A total RMSE value of 2303.31% and 773.17% of MAE are presented. There is a clear difference, and the magnitude order of the error perfectly defines a degraded dynamic behavior of the bridge. Based on expert recommendations, a limit of 50% for the MAE predicted values could be good to start thinking about degraded conditions in the infrastructure if such a limit is breached in most of the predicted samples. An accurate definition of the error thresholds corresponding to the health status of the structure requires a period of at least 12 months.

**Figure 20:**
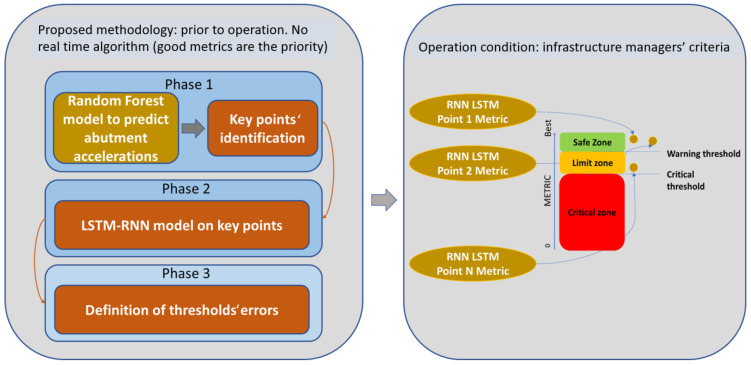
Asset monitoring model.

The different phases of this methodology are presented in Figure 20.

The threshold values are reached once the comparison between the prediction and the actual measurement is completed. These predictions are generated if extreme climatic conditions or a train passing over the bridge are detected. Consequently, real-time processing is not needed. Once the alerts are received by the infrastructure manager and the operator, the restrictions and mitigations are determined to guarantee a reliable and safe operation.

As the speed limitation is around 100 km/h, if degraded conditions are detected, it is recommendable to limit the train speed to 50 km/h so the driver can break before the bridge if water accumulation or river flooding is detected. This limit could be adapted depending on the trains and the brake efforts (directly related to the necessary brake distance). Such limitations on speed should be the result of the infrastructure manager’s conclusion after an analysis of the track quality [24] statement according to its maintenance processes.

From a technical point of view, it is important to highlight the following contributions to the state-of-the-art:

- The three major areas of artificial intelligence have been combined into a single methodology: computational perception by using sensors, machine learning, and deep learning [25,26,27].
- The Random Forest technique allows for the prediction of the structural response by monitoring only the critical points of the infrastructure.
- Random Forest algorithms enable critical points in the structure to be extracted, minimizing the number of accelerometers that need to be installed [28].
- LSTM recurrent neural networks [29] allow for the prediction of the response of the structure from a single accelerometer, decoupling measurements between sensors [30]. The failure of one sensor will not affect the measurements of the rest. Accelerometers are placed at the critical points identified by the SHAP algorithm, and each one predicts the response of a single critical point. An RNN is generated for each accelerometer. In case of an error in one of them, the comparison with the rest of the measurements will conclude the failure of that sensor, avoiding a false positive situation of a degraded condition of the bridge.

Considering the railway sector, the main contributions are exposed:

- A condition-based maintenance model is created with the aim of predicting the degraded condition of a bridge. This contributes to increasing safe and reliable operations.
- The proposed methodology demonstrates the efficiency of implementing artificial intelligence models for predictive maintenance.
- Reliability, safety, and maintainability of the infrastructure and the global railway operation are optimized, reducing economic and environmental costs.
- This work is included in the global action plan of Agenda 2030, with the aim of reducing emissions and energy consumption.

As a future work, some recommendations are made:

- Create a midterm plan for methodology validation in other infrastructures, determining more precisely the threshold values [19].
- Design a methodology for the sensing of new infrastructures with the aim of implementing, from the beginning, health status supervision in real-time. [31,32]
- Work on the definition of the risks and mitigations derived for the restrictive operation caused by degraded conditions of the infrastructure.
- Include information on the sensors installed in the rolling stock to complement the data generated in the infrastructure.
- Work on the implementation of a cloud service that coordinates with the rest of the safety facilities involved in the railway operation, which could allow coordinated decision-making.

Currently, regarding artificial intelligence models, infrastructure managers are implementing, along with continuous monitoring, the use of drones or unmanned aircraft systems (UASs) that allow the detection of degraded conditions that are not possible by visual human inspections due to being inaccessible or due to limitations of human vision [33,34,35]. It is interesting to combine both technologies according to their synergies in order to consolidate a holistic maintenance system.

## Figures and Tables

**Table 1:** Sampling parameters.

Nº Accelerometers	Nº Laser	Sampling Frequency (HZ)	Nyquist Frequency (Hz)
10	3	100	50

**Table 2:** Variables’ influence on the prediction of S1.

Predictor	Importance
2	0.637
7	0.18
8	0.046
6	0.0286
10	0.0241
5	0.0233
3	0.0232
9	0.0198
4	0.018

**Table 3:** RNN-LSTM architecture.

Parameter	Value
Batch Size	64
Layer 1	32 kernel filters
Layer 2	64 LSTM neurons
Layer 3	64 LSTM neurons
Layer 4	Dense layer with 30 neurons
Layer 5	Dense layer with 10 neurons
Output layer	1 neuron

**Table 4:** Training results of the RNN. % MAE performance indicators.

%MAE Acc.2	%MAE Acc.7	%MAE Acc.8
11%	9%	12%
11%		

**Table 5:** Safety criteria proposed.

Nº Acc. in Critical Zone	Nº Acc. in Limit Zone	Proposal
1	0	Immediate review of the transducer. Limit speed (LTV).
>2	Any	Circulation cancelled
0	1	Review of the transducer as soon as possible.
0	>2	Immediate review of the transducer. Limit speed (LTV).

**Table 6:** Comparison between the RMSE and MAE values obtained in different tested cases. Preliminary limit values proposal for degraded conditions.

	Normal Behavior	Not Normal Behavior	Limit Value
**RMSE**	0.8802	23.0331	1
**MAE**	0.3746	7.7317	0.5

## Data Availability

Data is unavailable due to privacy.
